# Comparison of Simoa, high‑sensitivity ELISA, and CLIA for serum neurofilament light chain quantification in multiple sclerosis

**DOI:** 10.1038/s41598-025-25926-0

**Published:** 2025-11-25

**Authors:** Kamila Zondra Revendova, Tereza Schaffartzikova, David Zeman, Pavel Hradilek, Pavlina Kusnierova

**Affiliations:** 1 Department of Neurology, University Hospital Ostrava, Ostrava, Czech Republic; 2 Department of Clinical Neurosciences, Faculty of Medicine, University of Ostrava, Ostrava, Czech Republic; 3 Institute of Laboratory Medicine, University Hospital Ostrava, Ostrava, Czech Republic; 4 Institute of Laboratory Medicine, Faculty of Medicine, University of Ostrava, Ostrava, Czech Republic

**Keywords:** Neurofilament light chain, Simoa, ELISA, CLIA, Multiple sclerosis, Biomarkers, Neurology, Neuroscience

## Abstract

Serum neurofilament light chain (sNfL) reflects neuro‑axonal injury, and is an emerging biomarker in multiple sclerosis (MS). This prospective cross-sectional study compared analytical agreement and clinical applicability among three analytical platforms. Serum samples from adult MS patients were analysed by single molecule array (Simoa) (frozen samples), high-sensitivity ELISA (hsELISA) (frozen samples), and fully automated chemiluminescent immunoassay (CLIA) (fresh and frozen samples). Simoa and hsELISA were strongly correlated (rₛ = 0.796) without systematic bias. CLIA with frozen samples showed higher sNfL levels (relative bias: 39.53% vs. Simoa; 29.56% vs. hsELISA). CLIA with fresh samples correlated very strongly with Simoa (rₛ = 0.820) and strongly with hsELISA (r_s_ = 0.764) (relative bias: −7.7% vs. Simoa; −14.3% vs. hsELISA), though broad limits of agreement indicated notable individual variability. Comparison of fresh vs. frozen CLIA values indicated an influence of pre-analytical conditions. Age was positively associated with sNfL determined by Simoa and hsELISA, whereas EDSS correlated only weakly with Simoa-derived sNfL. Simoa and hsELISA yield interchangeable sNfL results with consistent biological correlations, supporting their clinical and research application. CLIA can be automated but is affected by pre-analytical factors. Assay harmonization is essential before routine clinical implementation.

## Introduction

 Neurofilaments are major cytoskeletal proteins of neurons, which are most abundant in long projection axons. They comprise three main subunits that differ in molecular weight: light (NfL), medium, and heavy chains^1^. NfL chains are the most extensively studied, and are detected in cerebrospinal fluid (CSF) and peripheral blood during axonal damage^2^. Over the past decade, the development of ultrasensitive immunoassays has enabled the use of NfL as a clinically actionable biomarker—particularly in multiple sclerosis (MS), where there is a paramount need to detect subclinical disease activity and measure treatment efficacy. Elevated serum NfL (sNfL) levels have been detected in healthy individuals up to six years before the onset of clinical manifestations of MS^3^. In patients with MS, sNfL concentrations correlate with relapse and active lesions on magnetic resonance imaging (MRI), and predict risk of future disease activity and disease progression. Disease-modifying therapies (DMTs)—particularly monoclonal antibodies and, to a lesser extent, oral agents—tend to reduce sNfL concentrations to the levels observed in a normative reference population^4^.

Early applications of enzyme-linked immunosorbent assays (ELISAs) were sufficiently sensitive for NfL detection in CSF, but not in peripheral blood. The introduction of single molecule array (Simoa) technology constituted a major advancement that enabled reliable quantification of NfL at subpicogram concentrations in both serum and plasma^1^. Nowadays, Simoa is widely considered the gold standard for sNfL quantification owing to its ultra-sensitive detection and the extensive body of peer-reviewed literature confirming its validity and reproducibility. In parallel, the development of high-sensitivity ELISA (hsELISA) kits specifically optimized for blood matrices offered an alternative approach for blood-based NfL measurement^5^.

More recently, novel immunoanalytical techniques have emerged, including fully automated platforms suitable for routine clinical laboratories^6^. For example, the chemiluminescent immunoassay (CLIA) was developed by Siemens Healthineers (Atellica^®^ IM NfL), and offers picogram-level sensitivity, along with the benefits of automation. While Simoa is typically operated in batch mode, CLIA-based systems enable continuous access to testing, which may be advantageous in laboratories with higher sample volumes or that require immediate processing.

In the present study, the primary objective was to compare the analytical performance of hsELISA and CLIA with Simoa as a reference standard for sNfL quantification in a well-characterised MS cohort. Additionally, CLIA measurements from paired fresh and frozen serum samples were directly compared, to assess impact of storage condition. The secondary objective was to explore associations of sNfL concentrations with demographic and disease-related parameters in patients with relapsing-remitting MS (RRMS).

## Methods

### Study design and participants

This single-centre prospective cross-sectional study was conducted at the MS Centre, University Hospital Ostrava, Czech Republic. Patients were enrolled between June 4th and June 24th of 2025. The inclusion criteria were age $\:\ge\:\:$18 years; diagnosis of clinically isolated syndrome (CIS), radiologically isolated syndrome (RIS), or MS according to the latest 2017 revision of the McDonald criteria^7^; and providing signed informed consent to participate in the study.

### Clinical assessment and data

The following patient data were recorded: date of birth, sex, current DMTs, Expanded Disability Status Scale (EDSS) score at the time of sampling^8^, and occurrence of relapse within 3 months prior to sampling. Relapse was defined as a manifestation of any new or recurrent neurological symptoms lasting over 24 h, in the absence of fever or infectious disease^9^. EDSS and relapse occurrence were assessed by the treating MS specialist.

Ongoing DMTs for RRMS were categorized as low efficacy (all interferons, glatiramer acetate, teriflunomide, and dimethyl fumarate), moderate efficacy (cladribine, fingolimod, ponesimod, and ozanimod), or high efficacy (alemtuzumab, natalizumab, ocrelizumab, and ofatumumab). The high-efficacy treatment (HET) group also included patients who underwent immunoablative therapy, followed by autologous hematopoietic stem cell transplantation (AHSCT)^10^.

### Magnetic resonance imaging

Magnetic resonance imaging (MRI) scans of the brain and spinal cord were performed using 1.5T Magnetom Avanto Syngo B19 (Siemens, Erlangen, Germany) and 3 T Magnetom Prisma XA 30 (Siemens, Erlangen, Germany) MRI systems. The standard brain MS protocol included axial T2-weighted turbo spin echo (TSE) sequences; axial T2-weighted TSE fluid-attenuated inversion recovery (FLAIR) sequences; axial diffusion-weighted imaging with b-values of 0, 500, and 1000; and an automatically vendor-generated apparent diffusion coefficient map. All sequences were performed in 5-mm slices, with a 20% gap. Three-dimensional (3D) T1-weighted magnetization-prepared rapid gradient-echo, and 3D T2-weighted TSE sampling perfection with application-optimized contrasts using different flip angle evolution FLAIR sequences were performed, with isotropic acquisition in a 1-mm voxel, primarily in the sagittal plane.

For cervical spinal cord imaging, the protocol included sagittal T2-weighted TSE, axial T2-weighted gradient-recalled echo using multiple echo data image combination, and sagittal short tau inversion recovery T2-weighted TSE sequences. All images were acquired with a 3-mm slice thickness^11–14^.

MRI activity was evaluated during the year preceding sample collection. Disease activity was defined as the appearance of new T2 lesion/s or the enlargement of existing T2 lesion/s compared to previous scans, or presence of a gadolinium-enhancing lesion^15^.

### Samples

Serum samples were collected in tubes containing a clotting activator (Sarstedt). Upon delivery to the laboratory, serum samples were centrifuged at 2500 × g, for 6 min at 4 °C. Thereafter, the samples were distributed into four vials, with 0.5 mL allocated to each vial. One aliquot was promptly placed into an Attelica IM automatic analyser, and the Siemens method was used to ascertain sNfL. The remaining three aliquots were stored at − 80 °C until further analysis. All analyses of frozen samples were performed on the same day.

### Analytical methods

Serum NfL concentrations were measured using the following assays: the Simoa NF-light Advantage PLUS reagent kit (No. 104364, Simoa HD-X Analyzer; Quanterix; for research use only [RUO]), the NF-light™ (Neurofilament Light) Serum ELISA kit (No. 20–8002; UmanDiagnostics, a Quanterix company; RUO), and the Atellica^®^ IM Neurofilament Light Chain (NfL) assay (No. 11553991, Atellica IM; Siemens Healthcare; IVDR). According to the manufacturers, the lower limits of detection were 0.062 ng/L for the Simoa assay, 0.40 ng/L for the hsELISA assay, and 0.71 ng/L for the immunochemistry-based Atellica IM assay.

Whenever possible, the CLIA method (Siemens) was performed to analyse samples individually on the day of collection (*fresh samples*). CLIA accuracy was monitored using Atellica IM NfL Quality Control samples (NfL QC) from Siemens (< 3.9% CV). All sample analyses and QC were conducted within a two-week period. To assess the reproducibility of the CLIA test, three QC levels were analysed, generating 13 data points for each QC sample, yielding a total of 39 values. Intra-laboratory accuracy was calculated based on the CLSI 15-A2 analysis recommendation.

The remaining aliquots were subsequently frozen at − 80 °C by June 30th, 2025. On that date, all frozen aliquots were thawed simultaneously and re-analysed using the CLIA (*frozen*), Simoa, and hsELISA methods. The accuracy of the analysis was monitored using quality control samples included in the diagnostic kit from Quanterix (CV < 1.46%). The method’s reproducibility ranged from 7.94 to 9.42%, based on data obtained over three weeks, with 10 measurements at two levels. Concurrently, samples were analysed using the hsELISA method, which showed an accuracy of around 6.65%, and the CLIA method.

Creatinine was measured by enzymatic assay using an Atellica^®^ CH Analyzer with the Atellica CH Enzymatic Creatinine_2 (ECre_2) kit (Siemens Healthcare Diagnostics Inc.) The estimated glomerular filtration rate was calculated from serum creatinine, according to the CKD-EPI Equation^16^.

### Power calculation and statistical analysis

#### Power calculation

Before data collection, we performed a priori power analyses. For paired comparisons (paired t-test, two-sided α = 0.05, 1–β = 0.85), assuming a medium effect (Cohen’s *d = 0.50*), the required sample size was *N* = 38 pairs.

#### Statistical analysis

Statistical analyses were performed with MedCalc^®^ (version 22.021) and R software (version 4.2.0). The data were outlined using descriptive statistics. Categorical variables were evaluated using frequency tables. The distribution of continuous variables was visually assessed using histograms, and evaluated with the Shapiro–Wilk test. Data showing a normal distribution are reported as mean ± standard deviation (SD), while non‑normally distributed variables are reported as median and interquartile range (IQR). Associations between assays, and between CLIA-determined sNfL concentrations in fresh versus frozen samples, were assessed with Spearman’s rank correlation and Passing–Bablok regression. Systematic differences were examined by Bland–Altman analysis. Spearman’s correlation was calculated to explore relationships among age, EDSS, and sNfL concentrations in RRMS. The Wilcoxon rank‑sum (Mann–Whitney) test was used to evaluate differences in sNfL concentrations according to relapse occurrence or MRI activity. NfL concentrations were compared across DMT groups using the Kruskal–Wallis test, followed by Dunn’s pairwise test with Bonferroni adjustment. Spearman’s rank correlation coefficients were interpreted according to Prion and Haerling^17^. Statistical significance was set at *p* < 0.05.

## Results

This study included a total of 79 patients with MS, of whom 60 (75.95%) were women. Table 1 presents the demographic and clinical characteristics of the cohort.

**Table 1:** Demographic, laboratory, and clinical characteristics of the study participants.

Variable	
**Age**,** mean (SD)**	44.71 (10.46)
**Women**,** n (%)**	60 (75.95)
**sNfL concentration by Simoa**,** ng/L**,** median (IQR)**	8.39 (6.26–11.1)
**sNfL concentration by ELISA**,** ng/L**,** median (IQR)**	9.27 (7.49–12.50)
**sNfL concentration in frozen samples by CLIA**,** ng/L**,** median (IQR)**	13.04 (10.21–17.46)
**sNfL concentration in fresh samples by CLIA**,** ng/L**,** median (IQR)**, *n* = 56	7.67 (6.40–10.54)
**eGFR**,** ml/min/1.73 m**^**2**^, **median (IQR)**	104.4 (93.0–111.0)
**MS type**,** n (%)**	
**CIS**	1 (1.27)
**RIS**	2 (2.53)
**RRMS**	71 (89.87)
**PPMS**	2 (2.53)
**SPMS**	3 (3.80)
**EDSS**,** median (IQR)**	4.0 (2.0–5.0)

Abbreviations: CLIA = chemiluminescent immunoassay; CIS = clinically isolated syndrome; EDSS = Expanded Disability Status Scale; eGFR = estimated glomerular filtration rate; hsELISA = high-sensitivity enzyme-linked immunosorbent assay; IQR = interquartile range; SD = standard deviation; MS = multiple sclerosis; CIS = clinically isolated syndrome; PPMS = primary progressive multiple sclerosis; RIS = radiologically isolated syndrome; RRMS = relapsing–remitting multiple sclerosis; Simoa = single molecule array; SPMS = secondary progressive multiple sclerosis.

### Comparison of Simoa, hsELISA, and CLIA for determining sNfL concentrations in frozen samples

The median sNfL concentration in frozen samples was 8.39 (IQR: 6.26–11.1) ng/L when measured by Simoa, 9.27 (IQR: 7.49–12.50) ng/L by hsELISA, and 13.04 (IQR: 10.21–17.46) ng/L by CLIA. A strong correlation was observed between hsELISA and Simoa (r_s_ = 0.796, *p* < 0.001), a moderate correlation between CLIA and Simoa (r_s_ = 0.459, *p* < 0.001), and a weak correlation between CLIA and hsELISA (r_s_ = 0.376, *p* = 0.001).

**Fig. 1:**
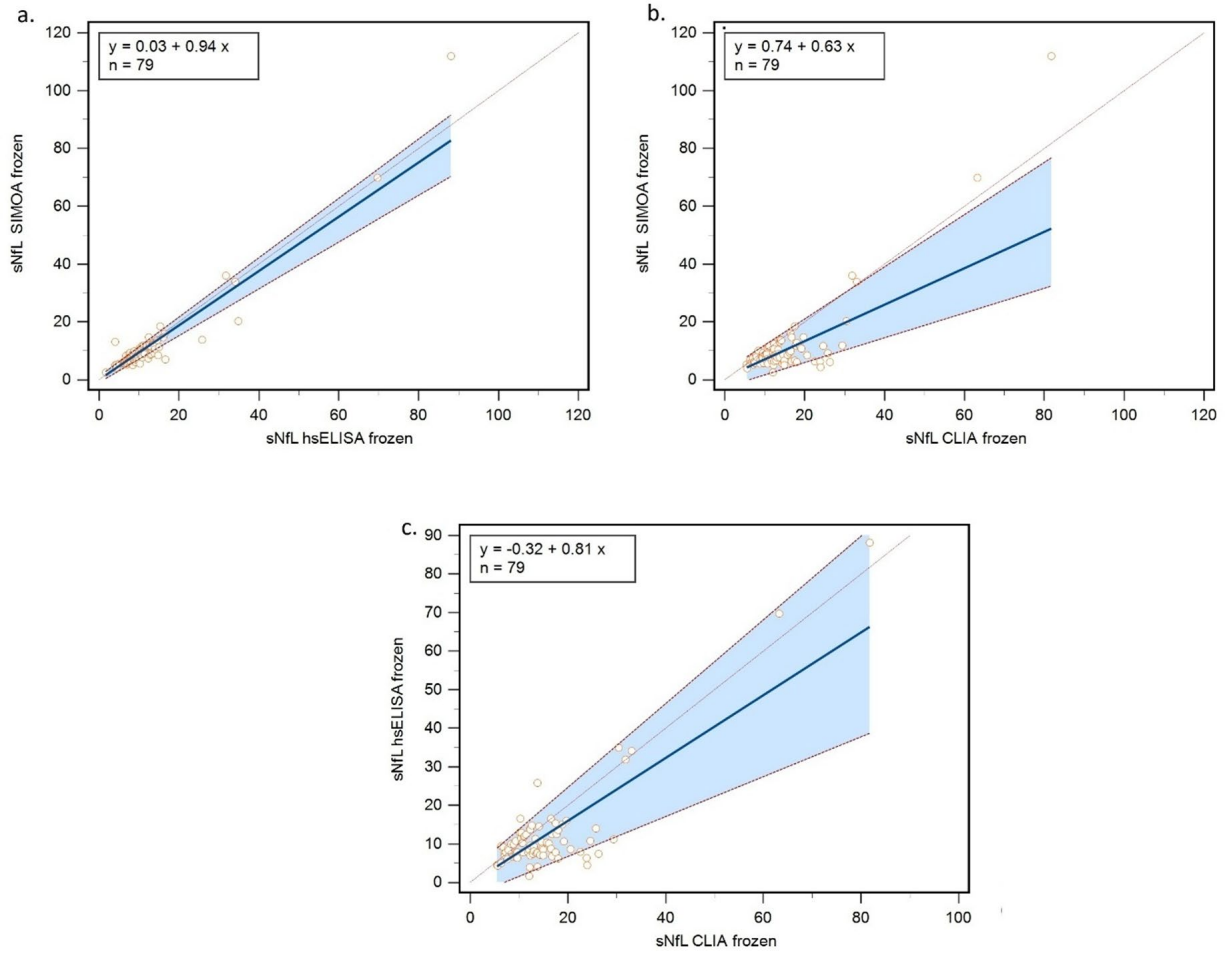
Passing–Bablok regression of (**a**) hsELISA vs. Simoa, (**b**) CLIA frozen vs. Simoa, and (**c**) CLIA frozen vs. hsELISA. Abbreviations: hsELISA = high-sensitivity enzyme-linked immunosorbent assay; Simoa = single molecule array; CLIA = chemiluminescent immunoassay; sNfL = serum neurofilament light chain.

Passing–Bablok regression revealed no significant difference between hsELISA and Simoa, with an intercept of 0.03 (95% CI: −0.94 to 1.12) and a slope of 0.94 (95% CI: 0.81 to 1.03). In contrast, comparison between CLIA and Simoa revealed a significant proportional difference, with a slope of 0.63 (95% CI: 0.43 to 0.90), and no systematic bias (intercept: 0.74, 95% CI: −2.61 to 2.95). No statistically significant difference was observed between CLIA and hsELISA (intercept: − 0.32, 95% CI: −3.62 to 2.92; slope: 0.81, 95% CI: 0.52 to 1.09) (Fig. 1).

**Fig. 2:**
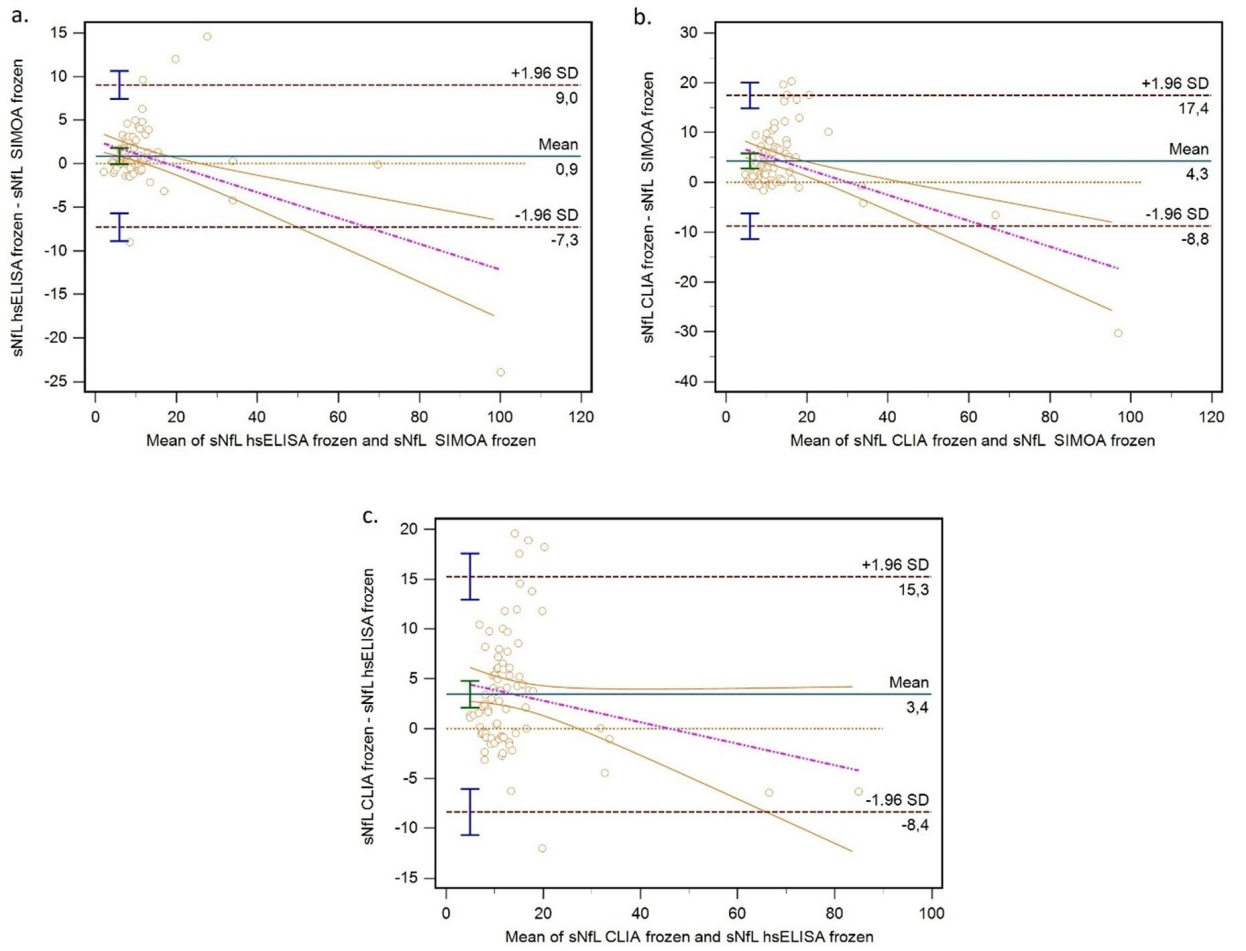
Bland–Altman plots of (**a**) hsELISA vs. Simoa, (**b**) CLIA frozen vs. Simoa, and (**c**) CLIA frozen vs. hsELISA. Limits of agreement are illustrated, and data are presented as ng/L. Abbreviations: hsELISA = high-sensitivity enzyme-linked immunosorbent assay; Simoa = single molecule array; CLIA = chemiluminescent immunoassay; sNfL = serum neurofilament light chain.

Bland–Altman analysis showed a mean difference between hsELISA and Simoa of 0.88 ng/L (95% CI: −0.06 to 1.81). The 95% CI of bias included zero, indicating negligible systematic error. Expressed relatively, hsELISA overestimated sNfL by 10.08% compared to Simoa. In contrast, CLIA yielded systematically higher values compared to Simoa (bias: 4.32 ng/L, 95% CI: 2.82 to 5.82) and hsELISA (bias: 3.44 ng/L, 95% CI: 2.09 to 4.79), corresponding to relative bias values of 39.53% and 29.56%, respectively. Table 2 presents the full results of Bland–Altman analysis, including absolute and relative biases and limits of agreement (LoA), and Fig. 2 shows Bland–Altman plots.

**Table 2:** Bland–Altman analysis results of comparison between the three analytical systems—Simoa, hsELISA, and CLIA.

		Bias	95% CI of bias	ULoA	95% CI of ULoA	LLoA	95% CI of LLoA
**hsELISA frozen vs. Simoa frozen**	ng/L	0.88	−0.06 to to1.81	9.04	7.44 to 10.64	−7.29	−8.89 to − 5.69
	%	10.08	4.27 to 15.89	60.95	50.98 to 70.93	−40.79	−50.77 to − 30.81
**CLIA frozen vs. Simoa frozen**	ng/L	4.32	2.82 to 5.82	17.44	14.87 to 20.02	−8.81	−11.38 to − 6.24
	%	39.53	30.89 to 48.18	115.18	100.35 to 130.02	−36.12	−50.96 to − 21.28
**CLIA frozen vs. hsELISA frozen**	ng/L	3.44	2.09 to 4.79	15.26	12.94 to 17.58	−8.38	−10.69 to − 6.06
	%	29.56	19.78 to 39.34	115.15	98.36 to 131.93	−56.02	−72.80 to − 39.23
**CLIA fresh vs. Simoa frozen**	ng/L	−1.92	−3.69 to − 0.15	11.05	8.00 to 14.10	−14.89	−17.94 to − 11.85
	%	−7.74	−13.92 to − 1.56	37.50	26.88 to 48.13	−52.98	−63.61 to − 42.36
**CLIA fresh vs. hsELISA frozen**	ng/L	−2.47	−3.73 to − 1.22	6.68	4.53 to 8.84	−11.65	−13.80 to − 9.49
	%	−14.29	−22.53 to − 6.05	46.01	31.85 to 60.18	−74.60	−88.76 to − 60.43

Abbreviations: CI = confidence interval; CLIA = chemiluminescent immunoassay; hsELISA = high-sensitivity enzyme-linked immunosorbent assay; ULoA = upper limit of agreement; LLoA = lower limit of agreement; Simoa = single molecule array.

### Comparison of sNfL values detected with CLIA of fresh samples versus Simoa and HsELISA of frozen samples

A total of 56 paired fresh and frozen serum samples were analysed. The median sNfL concentration in fresh samples was 8.60 (IQR: 6.26–11.3) ng/L as measured by Simoa, 8.77 (IQR: 7.24–12.59) ng/L by hsELISA, and 7.67 ng/L (IQR: 6.40–10.54) by CLIA. Serum NfL levels showed a very strong correlation between CLIA and Simoa measurements (r_s_ = 0.820, *p* < 0.001), and a strong correlation between CLIA and hsELISA measurements (rs = 0.764, *p* < 0.001).

**Fig. 3:**
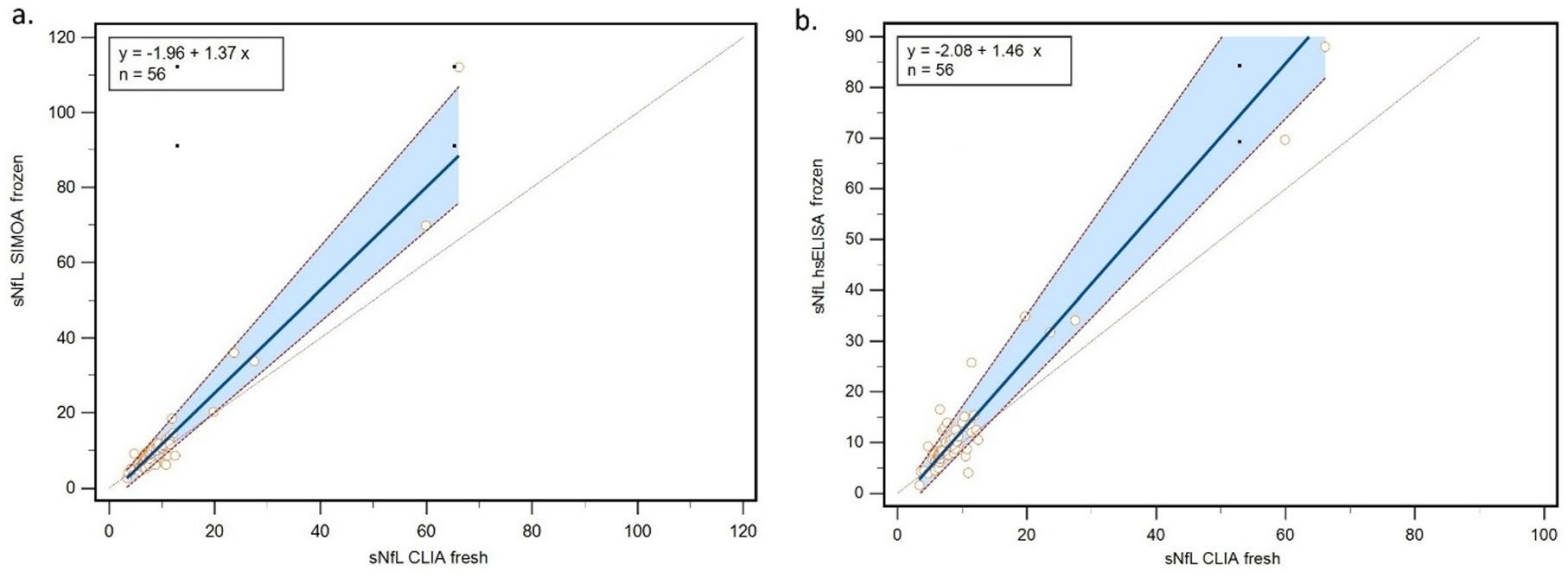
Passing–Bablok regression of (**a**) CLIA fresh vs. Simoa, and (**b**) CLIA fresh vs. hsELISA. Abbreviations: CLIA = chemiluminescent immunoassay; Simoa = single molecule array; hsELISA = high-sensitivity enzyme-linked immunosorbent assay; sNfL = serum neurofilament light chain.

Passing–Bablok regression analysis revealed significant systematic and proportional differences between CLIA and Simoa measurements, with an intercept of − 1.96 (95% CI: −3.86 to − 0.63), and a slope of 1.37 (95% CI: 1.21 to 1.63). Comparison between CLIA and hsELISA revealed an even greater proportional difference, with an intercept of − 2.08 (95% CI: −4.61 to − 0.98) and a slope of 1.46 (95% CI: 1.31 to 1.81). These findings indicated increasing differences with rising sNfL levels. For each 1 ng/L increase of sNfL as measured by CLIA, corresponding increases of 1.37 ng/L and 1.46 ng/L were observed in Simoa and hsELISA measurements, respectively (Fig. 3).

**Fig. 4:**
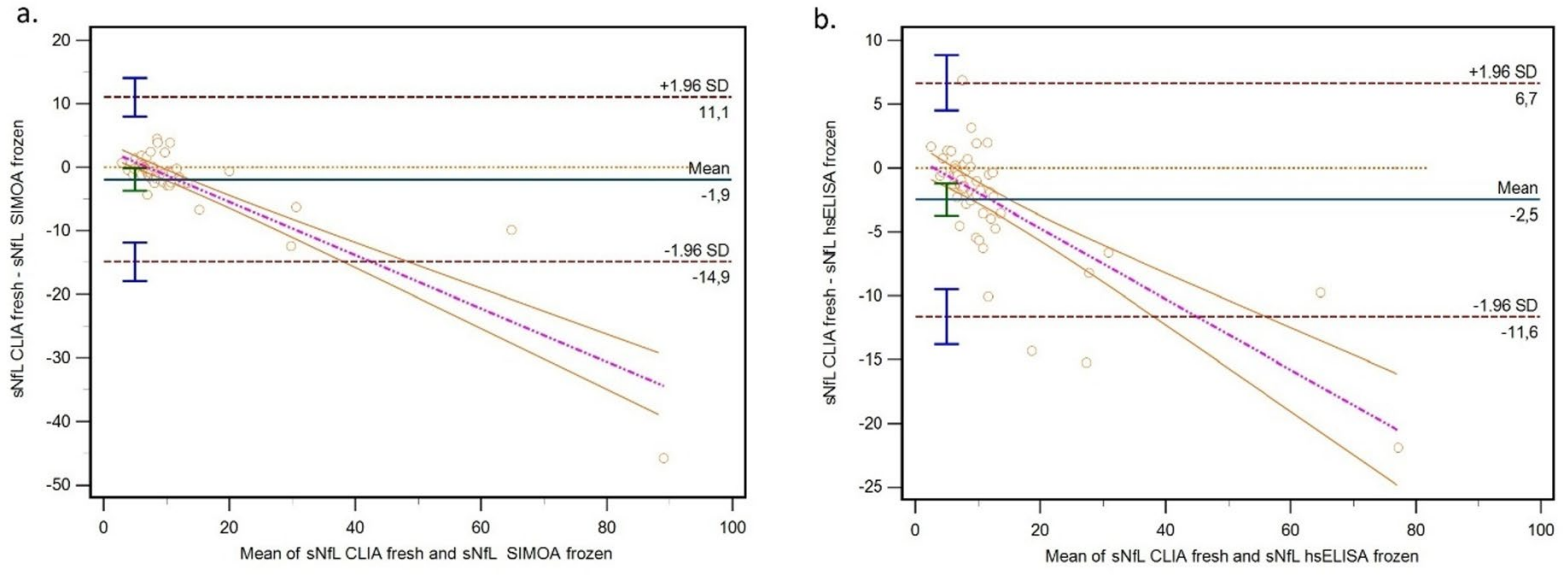
Bland–Altman plots of (**a**) CLIA fresh vs. Simoa, and (**b**) CLIA fresh vs. hsELISA. Limits of agreement are shown, and data are presented in ng/L. Abbreviations: CLIA = chemiluminescent immunoassay; Simoa = single molecule array; hsELISA = high-sensitivity enzyme-linked immunosorbent assay; sNfL = serum neurofilament light chain.

Bland–Altman analysis confirmed these discrepancies. CLIA showed a systematic underestimation compared to Simoa (mean bias: −1.92 ng/L, 95% CI: −3.69 to − 0.15) and compared to hsELISA (mean bias: −2.48 ng/L, 95% CI: −3.73 to − 1.22) (Table 2, Fig. 4).

### Differences between CLIA measurements of fresh versus frozen serum samples

**Fig. 5:**
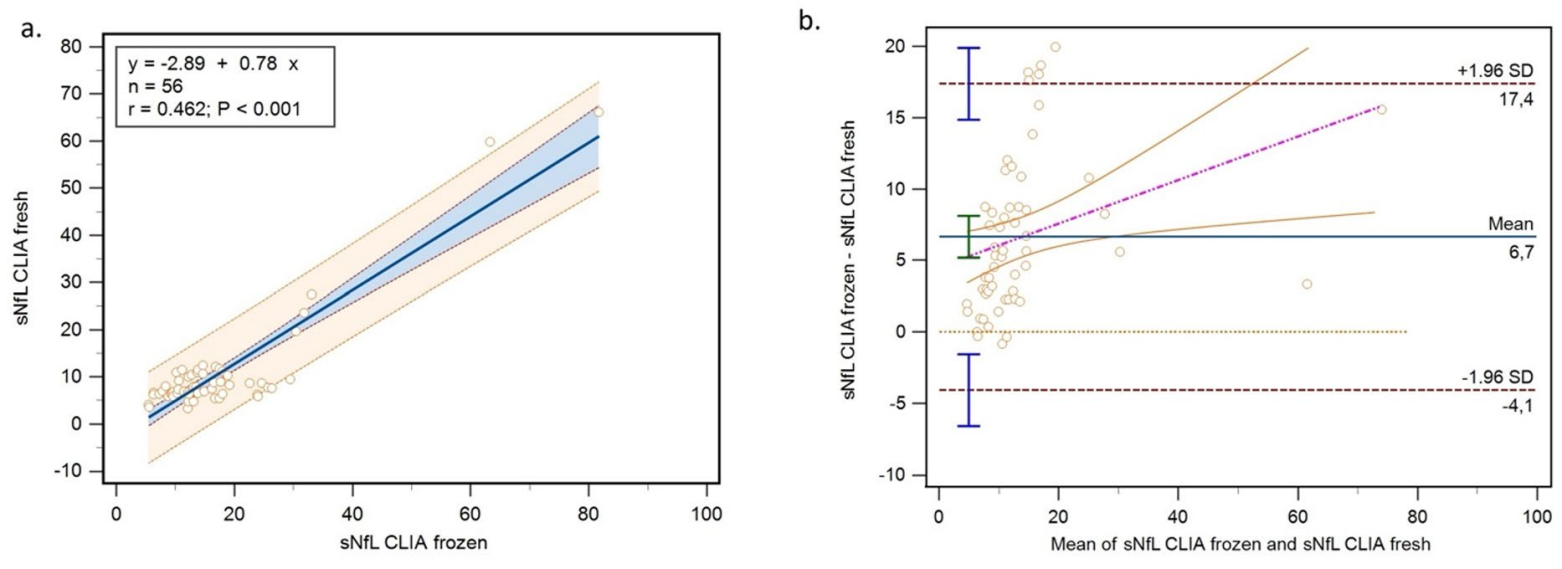
Comparison of sNfL concentrations measured by CLIA in fresh and frozen samples. (**a**) Passing–Bablok analysis. (**b**) Bland–Altman plots with limits of agreement. Abbreviations: sNfL = serum neurofilament light chain; CLIA = chemiluminescent immunoassay.

The sNfL concentrations measured by CLIA exhibited a moderate correlation between fresh and frozen samples (r_s_ = 0.462, *p* < 0.001) (Fig. 5a). Storage conditions (fresh vs. frozen) had a notable impact on sNfL concentrations assessed using the CLIA method, with an average difference of 50.7% (95% CI: −19.2% to 120.7%) and a median difference of 44.1% (95% CI: −4.82% to 117.5%) (Fig. 5b).

### Correlations of sNfL with demographic and disease-related parameters in RRMS

Age was correlated with sNfL when quantified by Simoa (r_s_ = 0.381, 95% CI 0.162 to 0.564, *p* = 0.001) and hsELISA (r_s_ = 0.323, 95% CI 0.097 to 0.517, *p* = 0.006), but not by CLIA from fresh samples (r_s_ = 0.209, 95% CI − 0.077 to 0.463, *p* = 0.151) or frozen samples (r_s_ = − 0.005, 95% CI − 0.237 to 0.229, *p* = 0.966). Median sNfL concentrations showed a significant sex-related difference, only when measured by CLIA from frozen samples (13.68 ng/L in women (95% CI 9.53 to 13.48) vs. 12.05 ng/L in men (95% CI 12.20 to 16.43), *p* = 0.033). EDSS showed a weak correlation with Simoa-derived sNfL (r_s_ = 0.252, 95% CI 0.019 to 0.458, *p* = 0.034), but no significant relationship with values obtained using hsELISA or CLIA. Across all assays, sNfL concentrations did not significantly differ between relapse vs. remission, with vs. without MRI activity, or among different DMT groups.

## Discussion

In this prospective cross-sectional study, we compared hsELISA and CLIA with Simoa, for quantification of the sNfL concentration in patients with MS. Our findings demonstrated a strong correlation between hsELISA and Simoa measurements (r_s_ = 0.796), with minimal bias in both Passing–Bablok and Bland–Altman analyses, supporting the analytical comparability of hsELISA with the digital Simoa platform. Notably, these results were markedly better than the comparison reported in our previous study^5^, which showed a bias of nearly 30%. In the present study, hsELISA overestimated Simoa values by an average of 10.08%, which is well within the clinically accepted error of ± 25%. Further supporting these findings, Pafiti et al.^18^ reported a similarly strong correlation (*r* = 0.919) between these two platforms.

On the other hand, the CLIA method used for frozen samples exhibited only a moderate correlation with Simoa (r_s_ = 0.459), and a weak correlation with hsELISA (r_s_ = 0.376). Bland–Altman analysis revealed substantial positive biases of 39.53% and 29.56%, respectively, which both exceed the acceptable limit of ± 25%, with very wide LoA. The CLIA method used for fresh samples exhibited better correlations with both Simoa (r_s_ = 0.820) and hsELISA (r_s_ = 0.764), yielding mean relative bias values of − 7.7% compared with Simoa, and − 14.3% compared with hsELISA. Nevertheless, the LoA remained broad (− 53.0% to 37.5% and − 74.6% to 46.0%), indicating the potential for sizeable deviations in individual samples. Storage conditions (fresh vs. frozen samples) had a pronounced effect on CLIA-derived sNfL, with an average difference of 50.7%. Our findings complement those of Lee et al.^19^, which demonstrated the analytical suitability of CLIA, and extend them by showing that serum handling (fresh vs. frozen) substantially affects CLIA results. Comparable data for hsELISA have not yet been published. By contrast, several studies have reported that Simoa-measured sNfL is largely stable under processing delays and after repeated freeze–thaw cycles, supporting the plausibility that Simoa is robust to freeze–thaw handling in blood matrices^20–22^.

These findings are in accordance with those of other recent studies evaluating automated laboratory platforms. For example, Booth et al.^23^ reported that although the Roche Elecsys NfL assay demonstrated excellent correlation with Simoa (*r* = 0.991), it significantly underestimated concentrations, showing a mean bias of − 85.1%. These results reinforce the notion that high correlation coefficients do not guarantee method interchangeability, and highlight the need to assess both systematic and proportional bias in method validation.

From a practical perspective, all three methods evaluated in our study offer picogram-level sensitivity that is suitable for blood-based NfL quantification. Despite its batch-based design, Simoa remains an efficient solution in clinical settings with low testing volume, and without a need for continuous on-demand analysis. In such settings, CLIA-based platforms may not be optimal because of the increased reagent consumption due to frequent calibrations and quality controls. Notably, our experience with the Atellica^®^ IM platform indicated a runtime of approximately 52 min per sample, reflecting a realistic processing time for automated assays in routine diagnostics.

This study was primarily designed for the analytical comparison of the three methods; nevertheless, we also explored clinical association with sNfL concentrations. Our results showed that age was correlated with sNfL levels measured from frozen samples by Simoa and hsELISA, which is in accordance with previous findings^6^. EDSS showed a significant but weak correlation with only Simoa-measured sNfL, in line with previous cross-sectional studies^2^. None of the three sNfL tests reliably discriminated between relapse and remission, or between patients with and without disease activity on MRI, most likely reflecting limited power given the cohort size and composition (most patients were treated with HET, and only 8 patients in our study exhibited disease activity on MRI in the preceding year), whereas larger studies have demonstrated higher sNfL concentration during clinical relapse and in presence of MRI activity^4,24,25^. Notably, for the routine monitoring of patients with MS, it is recommended to use age and body mass index (BMI) adjusted Z-scores or percentiles, or differences from the baseline level, rather than absolute values^26^.

One limitation of our study is the absence of a healthy control group, which would have enabled clearer interpretation of normal-range sNfL levels and assay-specific cut-offs. Additionally, we did not adjust for BMI, which is a known physiological factor influencing sNfL concentrations and thus could have affected absolute values and inter-individual variability. Other limitations were insufficient sample size for evaluation with clinical variables; therefore, these associations are only exploratory. In addition, CLIA was performed on fresh serum, whereas Simoa and hsELISA were run on frozen aliquots; although this reflects real-world use, it introduces a pre-analytical asymmetry.

Overall, findings corroborate the established role of Simoa as the gold standard for sNfL quantification due to ultra-sensitive detection and extensive validation, however hsELISA can be a reliable alternative to Simoa in clinical and research laboratories that lack access to digital immunoassay platforms. The fully automated CLIA platform offers practical advantages, but the results were affected by a sample storage. Analyses of frozen samples yielded an unacceptably large positive bias, whereas measurements from fresh samples fell within the ± 25% threshold but retained a wide LoA, suggesting considerable variability at the individual sample level. Future works should validate method-conversion equations in independent cohorts spanning a wider concentration range; rigorously characterise how storage conditions affect CLIA, and whether the fresh–frozen effect generalized to plasma and alternative tube types; and quantify the extent to which between-platform variability materially alters associations with clinical outcomes (relapses, MRI activity, and PIRA), to define the clinical relevance of analytical differences.

## Data Availability

The data that support the findings of this study are available from the corresponding author upon reasonable request.
