# Correction: Comparison of Simoa, high‑sensitivity ELISA, and CLIA for serum neurofilament light chain quantification in multiple sclerosis

**DOI:** 10.1038/s41598-025-32868-0

**Published:** 2025-12-18

**Authors:** Kamila Zondra Revendova, Tereza Schaffartzikova, David Zeman, Pavel Hradilek, Pavlina Kusnierova

**Affiliations:** 1 Department of Neurology, University Hospital Ostrava, Ostrava, Czech Republic; 2 Department of Clinical Neurosciences, Faculty of Medicine, University of Ostrava, Ostrava, Czech Republic; 3 Institute of Laboratory Medicine, University Hospital Ostrava, Ostrava, Czech Republic; 4 Institute of Laboratory Medicine, Faculty of Medicine, University of Ostrava, Ostrava, Czech Republic

Correction to: *Scientific Reports* 10.1038/s41598-025-25926-0, published online 25 November 2025

The original version of this Article contained an error in the spelling of the author Kamila Zondra Revendova which was incorrectly given as Kamila Zonda Revendova. The original Article has been corrected.

